# Early Transcriptome Response of *Trichoderma virens* to Colonization of Maize Roots

**DOI:** 10.3389/ffunb.2021.718557

**Published:** 2021-08-25

**Authors:** James T. Taylor, Ken-Der Wang, Benjamin Horwitz, Michael Kolomiets, Charles M. Kenerley

**Affiliations:** ^1^Department of Plant Pathology and Microbiology, Texas A&M University, College Station, TX, United States; ^2^Department of Biology, Technion Israel Institute of Technology, Haifa, Israel

**Keywords:** Trichoderma, transcriptome, maize, plant-microbe interaction, induced systematic resistance, SM1

## Abstract

*Trichoderma virens* is a well-known mycoparasitic fungal symbiont that is valued for its biocontrol capabilities. *T. virens* initiates a symbiotic relationship with a plant host through the colonization of its roots. To achieve colonization, the fungus must communicate with the host and evade its innate defenses. In this study, we explored the genes involved with the host communication and colonization process through transcriptomic profiling of the wild-type fungus and selected deletion mutants as they colonized maize roots. Transcriptome profiles of the *T. virens* colonization of maize roots over time revealed that 24 h post inoculation appeared to be a key time for plant-microbe communication, with many key gene categories, including signal transduction mechanisms and carbohydrate transport and metabolism, peaking in expression at this early colonization time point. The transcriptomic profiles of *Sm1* and *Sir1* deletion mutants in the presence of plants demonstrated that *Sir1*, rather than *Sm1*, appears to be the key regulator of the fungal response to maize, with 64% more unique differentially expressed genes compared to *Sm1*. Additionally, we developed a novel algorithm utilizing gene clustering and coexpression network analyses to select potential colonization-related gene targets for characterization. About 40% of the genes identified by the algorithm would have been missed using previous methods for selecting gene targets.

## Introduction

The rhizospheric fungus *Trichoderma virens* is well-known for its role as a plant symbiont that protects its host from pathogenic attack directly through mycoparasitism and indirectly through the induction of plant systemic defenses (Howell, 1987; Djonović et al., 2006a,b, 2007; Kubicek et al., 2011; Nawrocka and Małolepsza, 2013; Guzmán-Guzmán et al., 2017). This symbiotic relationship is perceived to be the result of fungal root colonization and communication with the host. There have been several studies that have described elements of the interaction between *T. virens* or other *Trichoderma* species and host plants (Brotman et al., 2008; Salas-Marina et al., 2011; Dubey et al., 2014; Crutcher et al., 2015; Lamdan et al., 2015; Morán-Diez et al., 2015; Nogueira-Lopez et al., 2018; Malinich et al., 2019). *T. virens* has been microscopically visualized extensively colonizing the external surface of maize roots, and internally colonizing a few cell layers deep before ceasing ingress (Nogueira-Lopez et al., 2018). Much of the initial penetration of the fungus is mediated by physical interactions and secretion of elicitor proteins and hydrophobins (Viterbo and Chet, 2006; Brotman et al., 2008, 2013; Crutcher et al., 2015; Gaderer et al., 2015). The expansin-like protein, Swollenin, was shown to have roles in both mechanical separation of plant cells to increase intercellular spaces and the plant recognition of a carbohydrate binding domain as a potential microbe-associated molecular pattern (MAMP; Brotman et al., 2008). *TasHyd1* is a hydrophobin that was demonstrated to be involved in the attachment of *T. asperellum* to cucumber roots, and when the gene was deleted, fungal colonization was significantly reduced compared to the wild-type strain (Viterbo and Chet, 2006). Additionally, a cerato-platanin family protein paralog of the elicitor protein SM1, designated SM2, was shown to be necessary for induced systemic resistance (ISR) against *Cochliobolus heterostrophus* and in colonization of maize roots (Crutcher et al., 2015; Gaderer et al., 2015). Internal colonization appears to be influenced by plant derived sucrose (Vargas et al., 2009, 2011). Vargas et al. found that mutant strains of *T. virens* lacking a key sucrose invertase colonized the roots at significantly greater levels than the wild type strain (Vargas et al., 2009).

Other transcriptomic studies have been performed to provide a greater understanding of the colonization process by *T. virens* (Morán-Diez et al., 2015; Malinich et al., 2019). These studies were conducted in hydroponic systems, but with significantly different timescales. Malinich et al. selected 6 and 30 h post inoculation (hpi) for an RNA-seq based transcriptomic study, while Moran-Diez et al. sampled at a single time point of 72 hpi for a microarray-based transcriptomic study. There were significant findings from each of these studies. Moran-Diez et al. explored the differences in *T. virens* gene expression in the presence of monocot and dicot hosts and Malinich et al. gave an in-depth analysis of the changes in *T. virens* gene transcription after the fungus recognizes a plant host and during colonization (6 and 30 hpi; Morán-Diez et al., 2015; Malinich et al., 2019). Despite the insights gleaned from these studies, the transcriptomic events of the very early stages of *T. virens* colonization (i.e., between 6 and 30 hpi) are not well-known. Additionally, the approach often used to select target genes for characterization and identification of their potential roles in plant-microbe interactions following analysis of transcriptomic data sets largely uses a “rack-and-stack” gene prioritization. This approach yields large lists of genes potentially involved in the colonization process that requires high throughput systems to identify those that are involved in plant-microbe interactions (Lamdan et al., 2015; Morán-Diez et al., 2015; Nogueira-Lopez et al., 2018). To further define the host-mediated response of maize to *T. virens* colonization, Wang et al. identified new oxylipin signals responsible for *T. virens* mediated induced systemic resistance by comparing the plant response to colonization by the wild-type strain, *Sm1* deletion mutant, or *Sir1* deletion mutant (Wang K. et al., 2020). SM1 is an elicitor required for the induced systemic resistance response mediated by *T. virens* (Djonović et al., 2006a, 2007). SIR1 is a small, secreted cysteine rich protein whose deletion increases the ISR response of colonized maize plants (Lamdan et al., 2015). However, comparatively little is known about the fungal response to the interaction with maize when these key genes are deleted.

Based on these identified gaps in knowledge and inefficient target selection methods, the following objectives were targeted: 1. Identify the early transcriptomic response of *T. virens* during the colonization of maize roots, 2. Create an efficient pipeline for analysis and target selection from transcriptomic data, and 3. Determine the influence of SM1 and SIR1 on the *T. virens* transcriptome during colonization.

To accomplish these objectives, we carried out an RNA-seq based transcriptomic study to explore the early events of colonization between 6 and 36 hpi. We developed a novel target enrichment pipeline that uses gene co-expression networks to select gene targets with higher likelihood of functional relevance to the interaction between the fungus and the plant and analyzed a previously sequenced transcriptomic dataset of the *T. virens* mutants Δ*Sm1* and Δ*Sir1* to compare to the transcriptome changes found in wild-type strain.

## Materials and Methods

### Strains and Hydroponic System

*T. virens* strain Gv29-8 (WT) was maintained on potato dextrose agar (PDA, BD Biosciences) at 27°C. *Zea mays* (Silver Queen hybrid, Burpee) was grown in a hydroponic system (Lamdan et al., 2015) as the host for the transcriptomic study with *T. virens*. The hydroponic system consisted of mason jars (500 ml, wide mouth, Ball, USA) with a four-pronged shaker clamp placed inside. Each jar was filled to the top of the clamp (~220 ml) with 0.5x Murashige-Skoog basal medium containing Gamborg's vitamins and supplemented with 0.5% sucrose. The unit was covered with a glass petri dish bottom and autoclaved. Plastic mesh (7 holes/linear inch) previously cut into discs to fit within the jars was autoclaved separately. After sterilization, the mesh discs were placed on top of the clamps, and pregerminated seeds with roots ~2 cm long were threaded through the mesh to contact the growth medium. The glass petri dish bottoms were replaced with sterile plastic petri dish bottoms to ensure a tighter fit.

### Colonization Assay

Following 3 days of incubation under lamps (Sun Blaze T5) with 6500K and 3000K lights at room temperature under a 14:10 light:dark regime with shaking at 50 rpm (New Brunswick Orbital Shaker), the roots of the seedlings were dipped in a suspension of 24-h-old *T. virens* mycelial tissue (4 g in 200 ml MS medium) for 30 s with gentle shaking. The inoculated roots were placed back into their original jars and returned to shaking under the light bank. Seedlings were harvested every 3 h for 12 h starting at 6 hpi. The harvested seedlings were gently rinsed in tap water and surface-sterilized for 1 min in 1% sodium hypochlorite, followed by a rinse for 1 min in sterile distilled water. Roots were detached from the seedlings and sliced into ~1 cm sections. The sections were plated on GVSM (Park et al., 1992, gliotoxin excluded) and incubated at 27°C for 24 h. The plates were observed for growth of the fungus originating from inside of the root.

### Transcriptomic Study

Hydroponic systems were established and roots inoculated as described previously. One gram of fungal tissue was used to inoculate hydroponic systems without plants to serve as controls. Colonized plant roots or fungal tissue cultured alone were harvested at 6, 12, 15, 24, and 36 h post inoculation, immediately flash frozen in liquid nitrogen, and stored for extraction. There were three biological replicates (5 plants pooled per biorep) per time point, giving a total of 30 samples.

### RNA Extraction and Preservation

The frozen roots were thoroughly ground in a mortar and pestle with liquid nitrogen. Approximately 500 mg of frozen powder was placed into a microfuge tube and 500 ul of TRI-reagent added. The mixture was allowed to fully thaw and vortexed to ensure homogenization of the sample. 500 ul of chloroform was added to the sample and vortexed to mix. The sample was then centrifuged for 10 min at 4°C and 14,000 rpm. The upper aqueous layer was collected in a new tube and one volume of ice-cold isopropanol added to precipitate the nucleic acids. The precipitant was spun in a microcentrifuge for 10 min at 4°C and maximum speed. The pellet was washed with 70% ethanol and allowed to dry before resuspending in 200 ul of nuclease free water. The sample was treated with DNAse I (Invitrogen, USA) as per manufacturer instructions. The sample was then electrophoresed on an agarose gel treated with 1% bleach to determine quality of the RNA and ensure that any DNA was degraded. The samples were then shipped on dry ice to the Technion Genome Center in Haifa, Israel, for sequencing.

### Analysis of Reads From RNA-Seq

Resulting raw reads for each sample were analyzed for read quality with fastQC software. All reads were then aligned with HISAT2 software and quantitated with StringTie. Differential expression analysis was performed in EdgeR. Differentially expressed genes (DEGs) were filtered to only those that had a SignalP score of 0.5 or higher. All scripts are available in the following github repository: (https://github.com/jamestaylorjr/Trichoderma_Transcriptome_Metabolome). The protein IDs used here reference the JGI protein ID. The JGI website (https://mycocosm.jgi.doe.gov/TriviGv29_8_2/TriviGv29_8_2.home.html) for *T. virens* proteins contains information on the transcript, location, predicted domains, and additional relevant information for each gene.

In addition to the differential expression analysis, the reads were normalized by the TMM method and exported as a separate file for clustering analysis. A co-expression network was generated using raw read counts from StringTie with the NetMiner software, with modifications to allow the use of StringTie formatted reads. The resulting co-expression network was used for further target gene selection.

### Gene Target Enrichment Pipeline

The Scikit-learn (sklearn) python library was used to perform principal component analysis on the TMM normalized read counts produced in previous steps of the analysis. The top three principal components were used as input to the sklearn DBSCAN algorithm for clustering. The generated co-expression network was queried for the centrality of each gene in each cluster using the betweenness centrality function in the Networkx python library. The most central gene in each cluster was selected as a putative target. Each unique gene in the list of putative targets was examined for putative function, domains, expression in other datasets, and putative subcellular localization.

### Transcriptomic Analysis of Δ*Sir1*/Δ*Sm1* Mutants

Reads from an RNA-seq transcriptomic experiment were obtained as in Malinich et al. (2019). Briefly, hydroponic systems were constructed with and without B73 plants and inoculated with one of three strains: Δ*Sm1* mutant, Δ*Sir1* mutant, or WT. At each time point there were four biological replicates of each mutant strain with and without seedlings, 10 biological replicates of the wild-type strain with seedlings, and four biological replicates of the wild-type strain without seedlings, for a total of 44 samples. Samples were collected at 6 and 30 hpi, RNA extracted, and sequenced. The reads were analyzed for differentially expressed genes as previously described, without filtering for the presence of a signal peptide.

## Results

### Selection of Timepoints for Transcriptomic Experiment

The colonization time-course experiment was performed to determine when the fungus begins its ingress of the roots. These data revealed that the fungus internally colonized as early as 12 h post inoculation. Replicate studies indicated that the fungus colonized ~50% of the samples at 12 hpi and consistently colonized the internal portion of all roots sampled at 15 hpi. Thus, we concluded that 12 hpi was a transitionary period in the colonization process, and 15 hpi represented the time of full ingress of the root in our system. To ensure immediate and consistent attachment of fungal inoculum to roots, our experimental set up employed a root dip method of inoculation. For transcriptome analysis, five time points were selected (6, 12, 15, 24, and 36 hpi) for tissue harvest that were considered to span the colonization process, including fungal-host communication.

### Analysis of Time-Course Transcriptomic Data

A quality check of sequenced reads was performed using fastqc, which demonstrated that the reads for all samples were of extremely high quality (>30 score). Alignment, quantification, and differential expression analysis were performed as described in the methods. As a previous study examined the general transcriptomic activity of *T. virens* in the presence of maize roots at 6 and 30 h post inoculation in great detail (Malinich et al., 2019), this study only focuses on the proteins that are predicted to be secreted. Filtering of DEGS to only those with a SignalP score >0.5 reduced the number of DEGs from 2,290 to 248. These genes were then grouped by the KOGG category to which they belong for comparison of up or downregulated genes across the time points. Only 8 KOGG categories were selected for further study due to having a high likelihood of involvement in plant-microbe interactions.

### Signal Transduction Mechanisms

**Figure 1:**
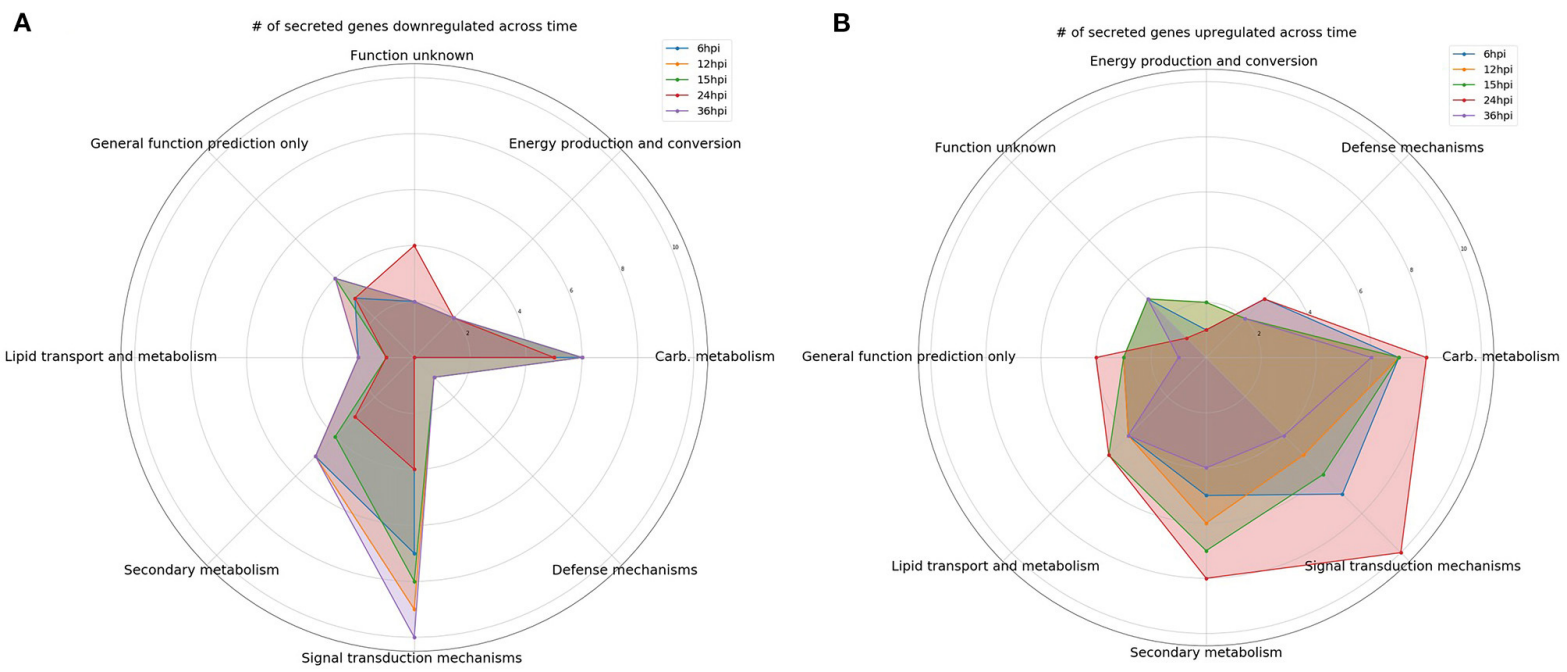
Comparison over time of the number of differentially expressed genes encoding predicted secreted proteins. Gene categories were selected based on perceived likelihood of involvement in the interaction between the fungus and plant. Each color corresponds to a different time point. **(A)** A radar plot of the number of downregulated genes over time. **(B)** A radar plot of the number of upregulated genes over time.

**Figure 2:**
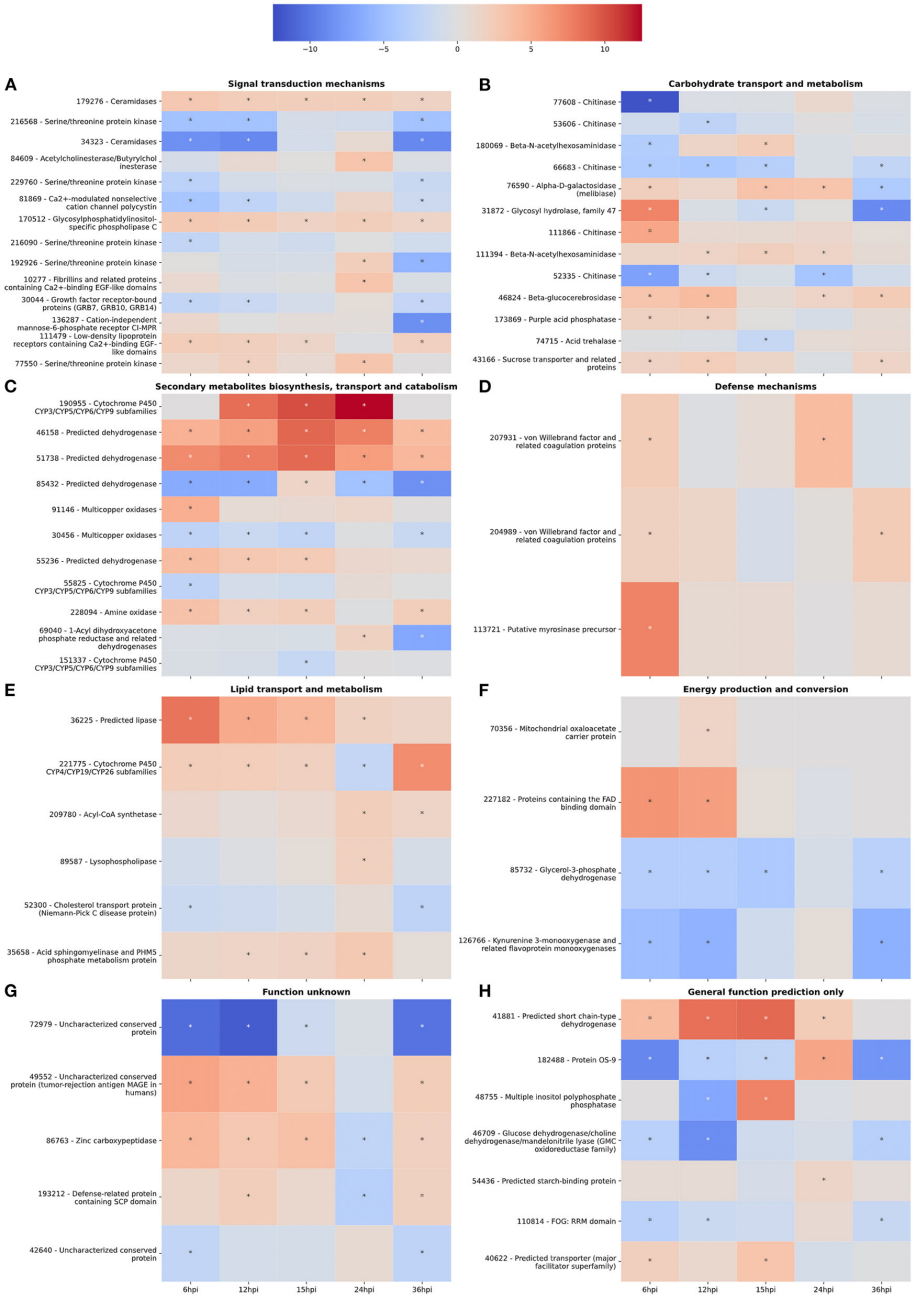
**(A–H)** Gene expression heatmaps of *T. virens* secreted proteins belonging to selected KOGG classes. KOGG classes were chosen based on potential involvement in the colonization process. The color scale of blue to red represents a range of −12.5 to 12.5 log2-fold change compared to the fungus cultivated alone at each corresponding time point. Asterisks denote an absolute value log2-fold change >1.5 and a false-discovery rate <0.05 as calculated during differential expression analysis in EdgeR.

Interestingly, there was an increase in the number of downregulated DEGs involved in signal transduction between 6 and 12 hpi (Figure 1A). However, the number of upregulated DEGs increased from 12 to 24 hpi before dropping off significantly at 36 hpi (Figure 1B). For example, gene 111479, encoding a putative chitosanase, was upregulated throughout the time course, except at 24 hpi where it was significantly downregulated (Figure 2A). Notably, there was a peak in expression of a ceramidase (34323) between 15 and 24 hpi, whereas a second ceramidase (179276) was upregulated throughout the interaction with the plant. Gene 10277, which contains the GLEYA motif associated with fungal adhesin proteins, was upregulated at 6 and 24 hpi. A putative CFEM domain gene (81869) was upregulated solely at 24 hpi. There were several serine/threonine protein kinases identified (192926, 77550, 216090, 216568, and 229760). Interestingly, one of these kinases (77550) also has a putative function as an alpha-1,3-glucan invertase based on interpro annotation.

### Carbohydrate Transport and Metabolism

At 6 hpi, there was significant downregulation of DEGs involved in carbohydrate transport and metabolism by the fungus (Figure 1A). The number of up and downregulated carbohydrate transport and metabolism genes remained largely unchanged until 24 hpi, when there was a spike in the number of upregulated genes followed by a drop, and corresponding spike in downregulated genes at 36 hpi (Figures 1A,B). There were five chitinases identified (111866, 77608, 52335, 53606, and 66683; Figure 2B). A LysM domain, found in chitin receptors, was present in 66683, which was largely downregulated during the interaction, but was induced at 24 hpi. Three of the five chitinases identified (52335, 111866, and 53606) belonged to the glycosyl hydrolase 18 family. Interestingly, these three chitinases showed different patterns of expression from one another. 111866 was mostly upregulated throughout the sampled times, whereas 52335 and 53606 were mostly downregulated. 43166, a putative sucrose transporter, showed upregulation at every time point except 24 hpi, at which it was downregulated.

### Secondary Metabolism

Intriguingly, the expression pattern of secondary metabolism related genes was similar to that of signal transduction mechanism genes, where the number of upregulated genes gradually increased between 6 and 24 hpi, followed by a sharp decrease (Figure 1B). DEGs in this category were largely dehydrogenases (46158, 51738, 85432, and 55236) and cytochrome P450s (151337, 55825, and 190955). There were also multicopper oxidases (91146 and 30456) and an amine oxidase (228094) that could be associated with nutrient scavenging (Figure 2C).

### Defense Mechanisms

DEGs associated with defense mechanisms remained largely upregulated throughout the time course (Figures 1A,B). The three DEGs in this category were two von Willebrand factor/coagulation proteins (207931 and 204989) and a putative myrosinase precursor (113721, Figure 2D).

### Lipid Transport and Metabolism

Lipid transport and metabolism related genes followed a similar pattern as defense mechanism related genes, with four upregulated DEGs at 6, 12, and 36 hpi, and five upregulated genes at 15 and 24 hpi (Figures 1A,B). Interestingly, one of the DEGs was a cytochrome P450 (221775), while the rest were lipases (36225 and 89587), cholesterol transport (52300), and other cell membrane associated enzymes (35658 and 209780; Figure 2E). The cytochrome P450, 221775, was moderately upregulated throughout the interaction, until 24 hpi, where it was moderately downregulated followed by strong induction at 36 hpi. 36225, a predicted lipase, was highly upregulated at 6 hpi, and remained upregulated throughout the time course, but with a lower magnitude as time passed.

### Energy Production and Conversion

Genes associated with energy production and conversion were largely downregulated across the time course (Figures 1A,B). At 6 and 12 hpi, a protein containing the FAD binding domain (227182) was highly upregulated, but expression returned roughly to that of the WT fungus alone from 15 to 36 hpi (Figure 2F). Another protein with a FAD binding domain (126766) showed a remarkably different expression pattern of downregulation throughout the interaction with a spike in expression at 24 hpi. 85732, a glycerol-3-phosphate dehydrogenase, showed a similar pattern of expression to 126766.

### General Function Prediction and Function Unknown

The genes in these categories have no known function or a very broad general function prediction. Their expression does not follow a consistent pattern (Figures 1A,B). However, these classes still contain interesting DEGs (Figures 2G,H). A notable DEG was a defense related protein containing the SCP domain (193212) that was upregulated at all time points except for 24 hpi, where it was significantly downregulated (Figure 2H). 72979 was highly downregulated at 6, 12, and 36 hpi, with a peak in expression at 24 hpi. This gene was annotated as a putative stress response protein. A predicted short chain dehydrogenase (41881) showed significant upregulation of ~10 log2-fold change at 12 and 15 hpi, which breaks from the pattern generally observed of a peak in differential expression at 24 hpi.

### Gene Network Analysis and Target Enrichment

A gene co-expression network was developed from the normalized read counts of the time course dataset to potentially identify “hub” genes with increased likelihood of a phenotypic effect in plant-microbe interactions. On its own, the co-expression network provides little insight due to its sheer size (Supplementary Figure 1). Therefore, a method of clustering secreted genes for correlation with the network was utilized. This method used a 3-dimensional principal component analysis matrix to cluster genes (Supplementary Figure 2). The generated co-expression network was queried for the centrality of each gene in each of the 18 clusters. The most central gene in each cluster was selected as a putative target for study of plant-fungus interactions.

### Target Selection From Clustering

Each unique gene in the list of putative targets was examined for putative function, domains, expression in other datasets, and putative subcellular localization (Table 1). One of the identified targets was the already characterized elicitor SM2 (111830). This protein has been demonstrated to have roles in both ISR and root colonization. Additionally, there are other putative targets that have a high likelihood of a large phenotypic impact due to predicted characteristics (i.e., secretion signals, gene ontology, KOGG categories, etc.) and previously reported experimental data. Six out of the 18 enriched targets were small, secreted cysteine-rich proteins (70780, 111642, 93159, 111830, 110875, and 58093), a trait commonly found in effector proteins. 50666 was previously isolated from the apoplast of maize plants treated with *T. virens*, though its function is unknown (Nogueira-Lopez et al., 2018). 214993 was found to contain a family 9 carbohydrate binding domain associated with cellulase activity. In addition to the method described, the network was queried for the gene with the highest degree, or largest number of connections. This revealed the previously identified, but uncharacterized gene *MRSP1*.

**Table 1:** List of enriched targets as determined by PCA, clustering, and centrality analysis.

**Gene**	**Domain**
TRIVIDRAFT_220864	NA
TRIVIDRAFT_70780	SSCP
TRIVIDRAFT_32983	Histidine phosphatase
TRIVIDRAFT_50666	Predicted bicupin
TRIVIDRAFT_60427	Ribonuclease
TRIVIDRAFT_111642	SSCP
TRIVIDRAFT_83765	NA
TRIVIDRAFT_93159	SSCP
TRIVIDRAFT_59198	RLP-like
TRIVIDRAFT_41881	Predicted short chain-type dehydrogenase
TRIVIDRAFT_111830	SM2 (known defense elicitor)
TRIVIDRAFT_110875	SSCP
TRIVIDRAFT_78354	Phospholipase A2
TRIVIDRAFT_70901	Polysaccharide lyase
TRIVIDRAFT_203083	NA
TRIVIDRAFT_58093	SSCP (Killer Toxin KP4)
TRIVIDRAFT_177054	S8 protease inhibitor
TRIVIDRAFT_214993	Contains carbohydrate binding domain

*Gene annotations and predictions were included as available. Each gene was queried through the JGI annotation database for T. virens. SSCP, small, secreted cysteine-rich protein*.

### Analysis of Δ*sm1*/Δ*sir1* Transcriptome Data

**Figure 3:**
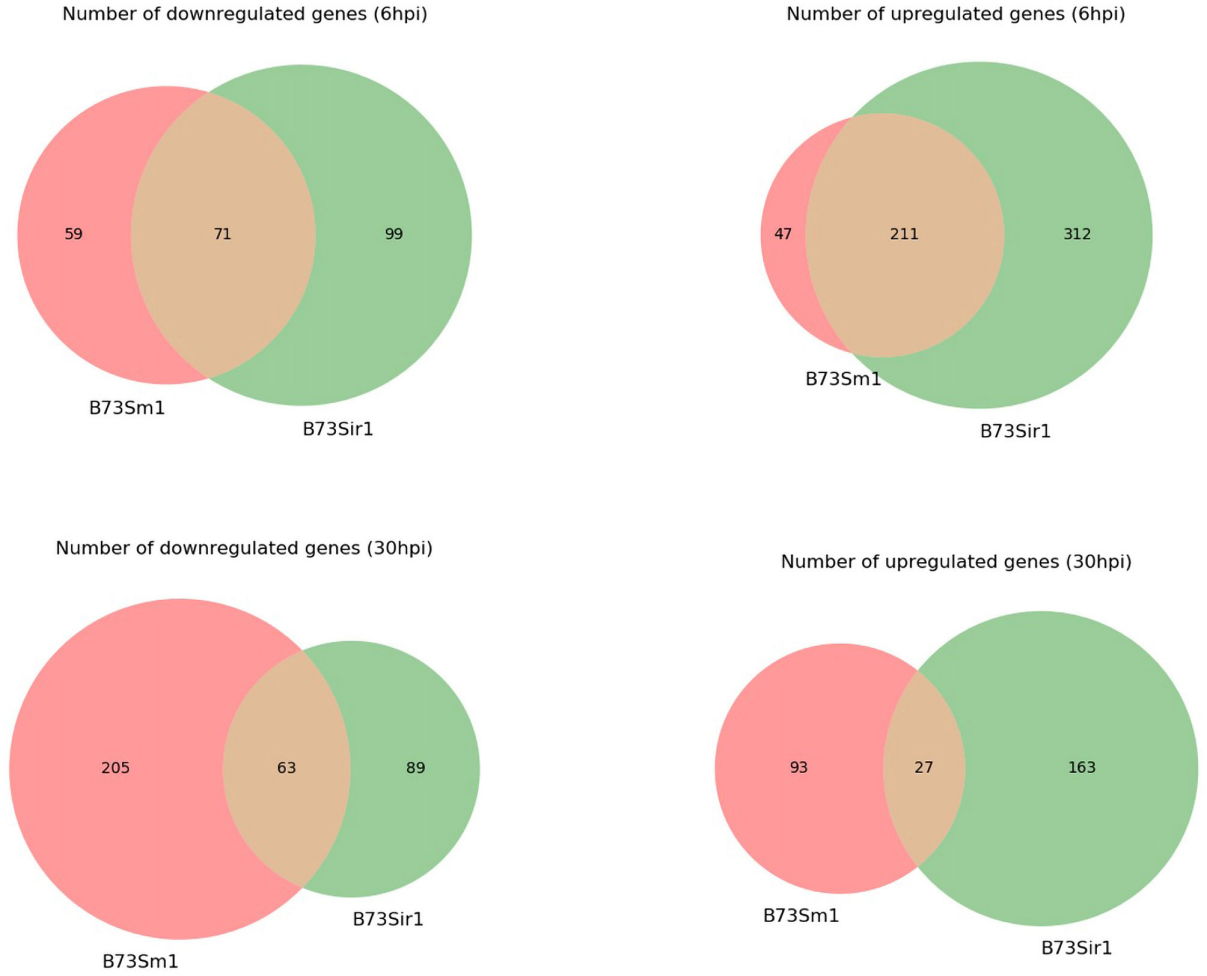
The number of unique genes differentially expressed in Δ*sm1* or Δ*sir1* mutants compared to the wild type strain in the presence of B73 maize roots at 6 and 30 hpi. The red portion of each Venn diagram corresponds to genes with unique expression patterns in Δ*sm1*, green corresponds to genes with unique expression patterns in Δ*sir1*, and orange corresponds to genes with similar expression patterns in both conditions.

**Figure 4:**
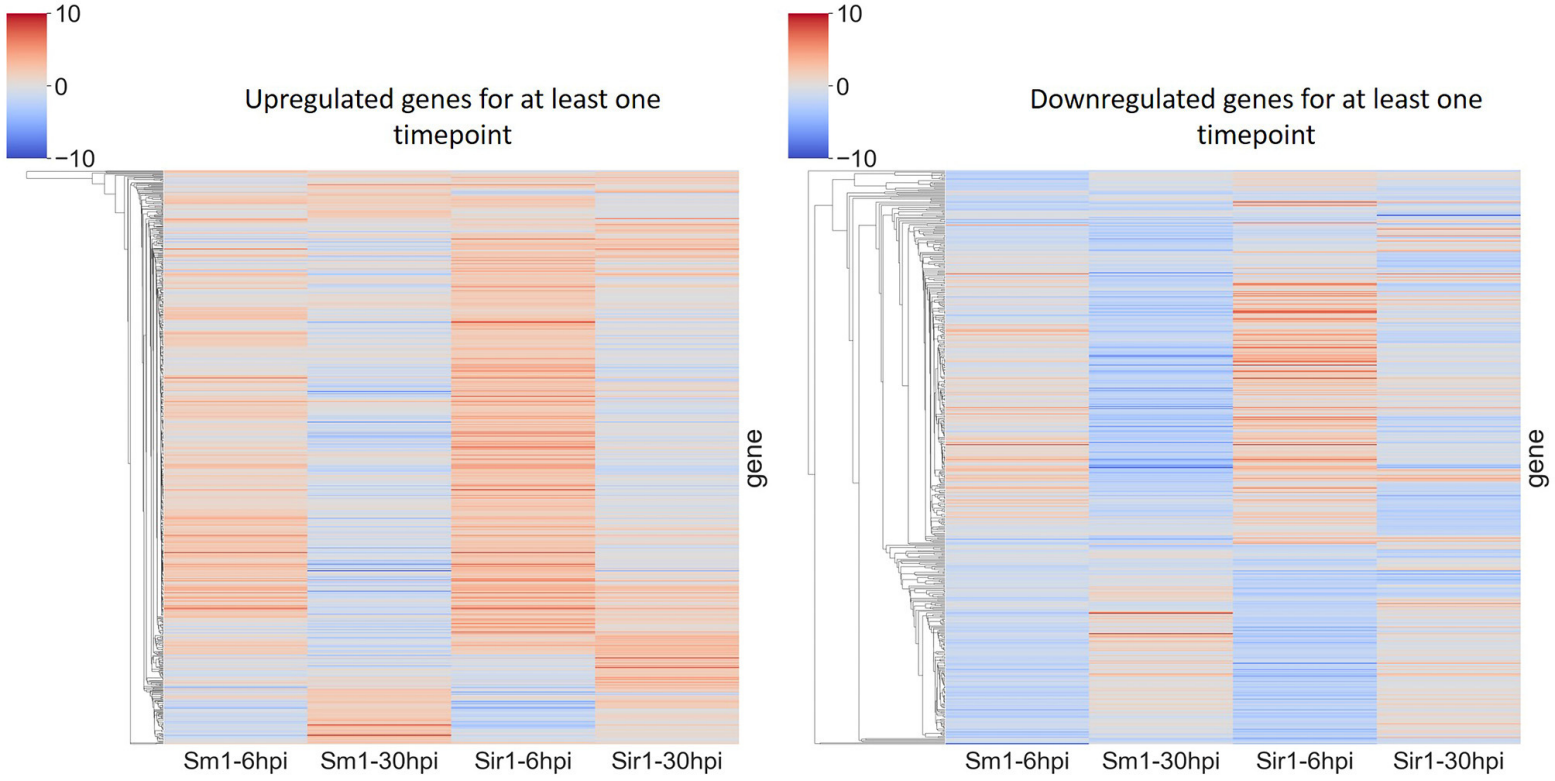
Heatmap comparison of differential gene expression from Δ*sm1* or Δ*sir1* mutants at 6 and 30 hpi. This comparison shows all genes that were differentially expressed during at least one time point. The color gradient from blue to red represents differential expression (log2-fold change), downregulation in blue and upregulation in red.

In addition to the time course dataset, an additional dataset from a previous experiment consisting of the transcriptomic response of *Sm1* and *Sir1* mutant strains in the presence of maize roots at 6 and 30 hpi was analyzed by differential expression analysis compared to the wild-type fungus in the presence of maize roots. Initial DEG analysis revealed a significantly larger number of unique up and downregulated genes in the *Sir1* mutant compared to the Δ*sm1* mutant at 6 hpi (Δ*sir1*: 312 and 99; Δ*sm1*: 47 and 59; Figure 3). Interestingly, there were a larger number of unique downregulated genes in the Δ*sm1* strain at 30 hpi (205 vs. 89 in Δ*sir1*), but Δ*sir1* had a larger number of unique upregulated genes at 30 hpi (163 vs. 93 in Δ*sm1*; Figure 3). Heatmap analysis of genes that were either up or downregulated for at least one timepoint showed that many of the genes that were upregulated at 6 hpi were downregulated at 30 hpi, and vice versa (Figure 4).

**Figure 5:**
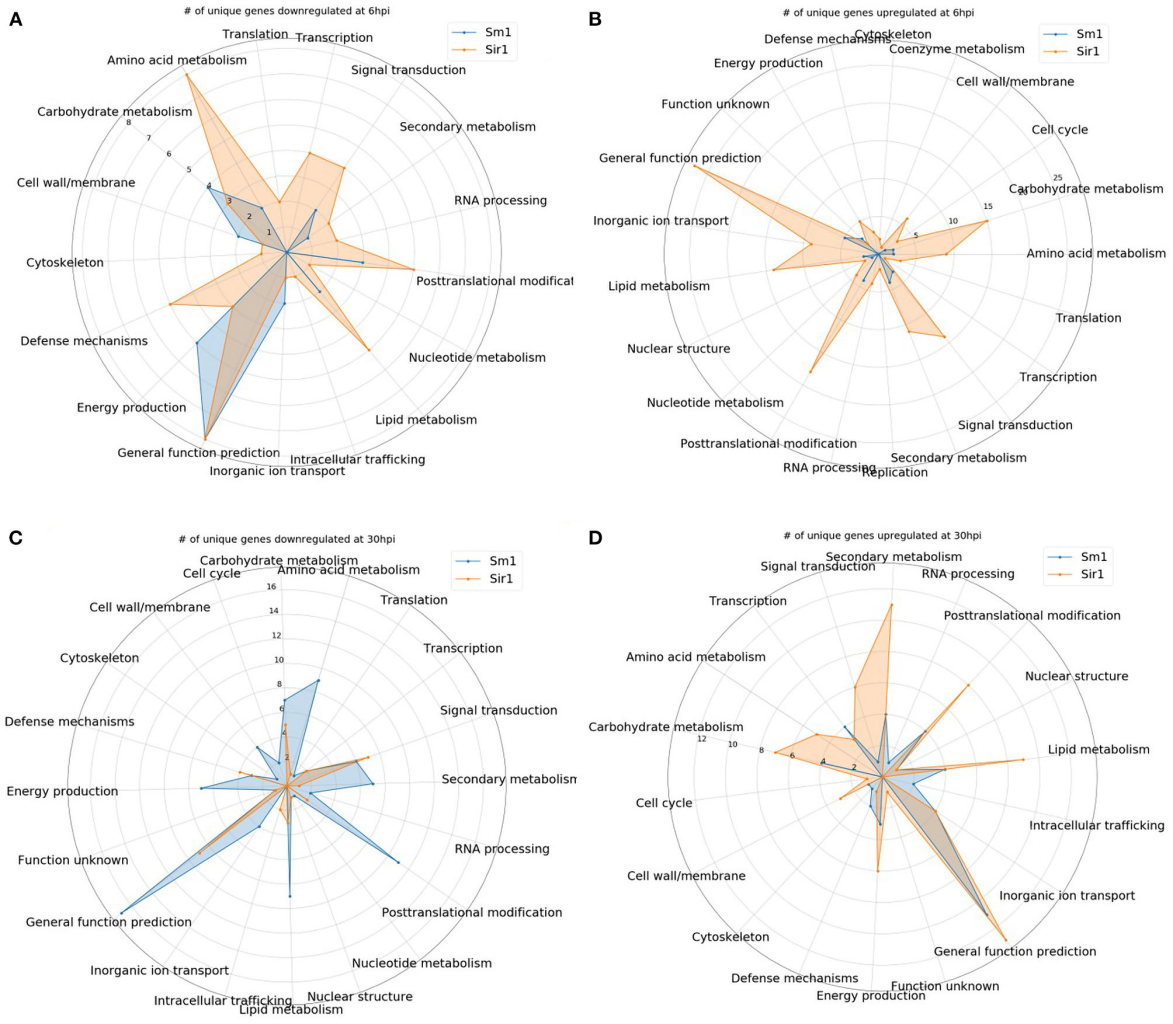
**(A–D)** The number of unique genes up or downregulated in each the Δ*sir1 and* Δ*sm1* strains at 6 or 30 hpi. Gene annotation categories were not filtered. Only those with at least one unique gene were included for comparison.

Many of the 6 hpi downregulated genes in the *Sir1* mutant were annotated as being involved in protein production and modification (Figure 5A). There was also a decrease in the genes categorized as signal transduction and defense mechanisms. The 6 hpi downregulated genes in the Δ*sm1* mutant mostly were involved in carbohydrate transport/metabolism and energy production (Figure 5A). Both mutants had a large number of genes that only had general function predictions.

In contrast to the genes that were downregulated in the Δ*sm1* mutant at 6 hpi, the Δ*sir1* mutant had a large number of carbohydrate transport/metabolism genes upregulated at 6 hpi (Figure 5B). There was also a significant number of signal transduction mechanism, secondary metabolism, and general function prediction genes upregulated at 6 hpi in the Δ*sir1* mutant.

The 30 hpi genes downregulated in the Δ*sm1* mutant was similar in both number and category to the Δ*sir1* mutant at 6 hpi: many genes involved in protein production and modifications (Figure 5C). The Δ*sir1* mutant displayed an increase in the number of signal transduction mechanism genes downregulated at 30 hpi (Figure 5C). Again, both mutants had a significant number of genes with only general function predictions downregulated at 30 hpi.

The pattern of upregulated Δ*sir1* mutant genes at 30 hpi is mostly similar to 6 hpi; however, there was a notable increase in the number of genes involved in energy production (Figure 5D). The Δ*sm1* mutant had a higher number of upregulated genes involved in transcription at this time point (Figure 5D). As with most of the other comparisons, there was a significant number of upregulated genes with general function predictions for each mutant at 30 hpi.

For all comparisons, there were a large number of unique genes with only general function predictions. These genes were examined in more detail. The majority of 6 hpi downregulated Δ*sir1* mutant genes were predicted to be major facilitator superfamily transporters, whereas the majority for the Δ*sm1* mutant were broad substrate reductases (Table 2). The Δ*sir1* mutant had a large number of predicted zinc-finger proteins upregulated at 6 hpi. In addition, there were many metal-dependent hydrolases, predicted hydrolases, and major facilitator superfamily transporters also upregulated in the Δ*sir1* mutant (Table 3). There were very few unique Δ*sm1* genes upregulated at 6 hpi, and no discernable pattern to the annotations (Table 3).

**Table 2:** Downregulated genes with general function KOGG annotation in Δ*sm1* or Δ*sir1* at 6 hpi.

**Downregulated genes 6 hpi**	**# DEGs in category**	
**Predicted function**	**Δsm1**	**Δsir1**
Aldehyde reductase	1	0
Aldo/keto reductase family proteins	1	0
FOG: Ankyrin repeat	1	1
FOG: RRM domain	0	1
Glucose dehydrogenase/choline dehydrogenase/mandelonitrile lyase (GMC oxidoreductase family)	0	1
Predicted dehydrogenase	1	0
Predicted fumarylacetoacetate hydralase	1	0
Predicted transporter (major facilitator superfamily)	1	3
Reductases with broad range of substrate specificities	2	1
Synaptic vesicle transporter SVOP and related transporters (major facilitator superfamily)	0	1

*Predicted functions were derived from GO terminology or JGI annotations*.

**Table 3:** Upregulated genes with general function KOGG annotation in Δ*sm1* or Δ*sir1* at 6 hpi.

**Upregulated genes 6 hpi**	**# DEGs in category**	
**Predicted function**	**Δsm1**	**Δsir1**
Aldehyde reductase	0	1
C4-type Zn-finger protein	0	1
FOG: Zn-finger	0	4
Glucose dehydrogenase/choline dehydrogenase/mandelonitrile lyase (GMC oxidoreductase family)	0	1
Glyoxylase	0	1
Hydroxyindole-O-methyltransferase and related SAM-dependent methyltransferases	1	0
Large RNA-binding protein (RRM superfamily)	0	1
Monodehydroascorbate/ferredoxin reductase	0	1
Multifunctional pyrimidine synthesis protein CAD (includes carbamoyl-phophate synthetase, aspartate transcarbamylase, and glutamine amidotransferase)	1	0
Predicted Zn-finger protein	0	1
Predicted fumarylacetoacetate hydralase	0	1
Predicted hydrolases or acyltransferases (alpha/beta hydrolase superfamily)	0	2
Predicted kinase	0	1
Predicted metal-dependent hydrolase of the TIM-barrel fold	0	3
Predicted short chain-type dehydrogenase	1	0
Predicted transporter (major facilitator superfamily)	0	3
Reductases with broad range of substrate specificities	1	2
Synaptic vesicle transporter SV2 (major facilitator superfamily)	0	1
Synaptic vesicle transporter SVOP and related transporters (major facilitator superfamily)	1	2
WD40 repeat-containing protein	0	1

*Predicted functions were derived from GO terminology or JGI annotations*.

The Δ*sm1* mutants had a significant number of predicted transporters and other metabolism related genes that were downregulated at 30 hpi (Table 4). The Δ*sir1* mutants followed a similar pattern, but to a lesser degree (Table 4).

**Table 4:** Downregulated genes with general function KOGG annotation in Δ*sm1* or Δ*sir1* at 30 hpi.

**Downregulated genes 30 hpi**	**# DEGs in category**	
**Predicted function**	**Δsm1**	**Δsir1**
Acetyltransferase, (GNAT) family	1	0
FAD-dependent oxidoreductase	1	0
FOG: Ankyrin repeat	0	1
FOG: RRM domain	0	1
FOG: Transposon-encoded proteins with TYA, reverse transcriptase, integrase domains in various combinations	1	0
FOG: Zn-finger	0	1
Glucose dehydrogenase/choline dehydrogenase/mandelonitrile lyase (GMC oxidoreductase family)	2	1
Hydroxyindole-O-methyltransferase and related SAM-dependent methyltransferases	1	0
Metallopeptidase	1	0
Predicted ATPase	0	1
Predicted NAD-dependent oxidoreductase	1	0
Predicted metal-dependent hydrolase of the TIM-barrel fold	1	0
Predicted steroid reductase	2	0
Predicted transporter (major facilitator superfamily)	3	2
Reductases with broad range of substrate specificities	1	1
Synaptic vesicle transporter SVOP and related transporters (major facilitator superfamily)	2	1

*Predicted functions were derived from GO terminology or JGI annotations*.

Genes that were upregulated at 30 hpi for both Δ*sm1* and Δ*sir1* were mostly annotated as transporters of different varieties (Table 5). The Δ*sir1* mutants also had several hydrolases and methyltransferases upregulated at this time point (Table 5).

**Table 5:** Upregulated genes with general function KOGG annotation in Δ*sm1* or Δ*sir1* at 30 hpi.

**Upregulated genes 30 hpi**	**# DEGs in category**	
**Predicted function**	**Δsm1**	**Δsir1**
C2H2-type Zn-finger protein	1	0
FOG: Ankyrin repeat	1	0
FOG: Zn-finger	1	0
Hydroxyindole-O-methyltransferase and related SAM-dependent methyltransferases	0	2
Large RNA-binding protein (RRM superfamily)	1	0
Multifunctional pyrimidine synthesis protein CAD (includes carbamoyl-phophate synthetase, aspartate transcarbamylase, and glutamine amidotransferase)	1	0
Permease of the major facilitator superfamily	1	0
Predicted haloacid-halidohydrolase and related hydrolases	0	1
Predicted short chain-type dehydrogenase	0	3
Predicted transporter (major facilitator superfamily)	2	4
Reductases with broad range of substrate specificities	1	1
Synaptic vesicle transporter SVOP and related transporters (major facilitator superfamily)	2	2

*Predicted functions were derived from GO terminology or JGI annotations*.

## Discussion

There have been several studies that sought to elucidate fungal transcriptome or proteome during the colonization process in a hydroponic setting (Lamdan et al., 2015; Morán-Diez et al., 2015; Nogueira-Lopez et al., 2018). All of these studies estimated the colonization process (i.e., growth and ingress within the root) required longer than 24 h to occur. Our results demonstrate that internal colonization in a setting that favors fungal growth can occur as early as 12 hpi, but more completely by 15 hpi. By bracketing the time points of a transcriptomic study around 15 hpi, an unprecedented level of detail of the colonization process was captured.

Malinich et al. analyzed in detail the broad patterns of gene expression exhibited by *T. virens* in the presence of maize (Malinich et al., 2019). This study focused on identifying a subset of genes involved in the interactions between *T. virens* and maize by filtering the differentially expressed genes based on secretion signal predictions and functional annotation and at a broader range of time points encompassing the early fungus-plant recognition events (6 hpi) and different stages of colonization (12, 15, 24, and 36 hpi).

The upregulation of the genes involved in signal transduction appears to coincide with increased fungal colonization. The noted drop in upregulation at 36 hpi may indicate that penetration and maximum growth within the root system has occurred. One of the genes that had a peak between 15 and 24 hpi was annotated as a ceramidase. Ceramidases catalyze the breakdown of ceramide into sphingosine, that, in addition to being a precursor to sphingolipids, may have its own signaling activities (Li et al., 2015). Sphingolipids have roles in signaling and cellular organization (Michaelson et al., 2016). Expression of the putative adhesin gene, 10277, suggests that it may be aiding the attachment of hyphae to plant cell walls. GLEYA domain containing proteins are known to bind to carbohydrates which adds support to this hypothesis (Willaert, 2018). The expression of other known signal transduction mechanisms increased up to 24 hpi, after which expression dropped off significantly. This suggests that the time between the initiation of hyphal penetration and expansion is critical for plant-fungal interaction, whereas signal communication seems to decrease after 24 hpi. This conclusion is supported by the expression pattern of a putative CFEM domain protein, 81869. CFEM domain proteins are commonly associated with virulence in pathogenic fungi, however, in the case of *T. virens* they are likely to be involved in colonization (Zhu et al., 2017).

The pattern of carbohydrate transport and metabolism gene expression shows an interesting trend of low numbers of upregulated genes prior to a spike only at 24 hpi. The 24 hpi spike and 36 hpi downregulation coincides with the peak in signaling related genes. Interestingly, the majority of the DEGs in this category were chitinases. One of these chitinases contained a LysM domain (66683). Based on its expression pattern and domain annotation, this protein is likely involved in prevention of the plant MAMP response to chitin. The other chitinases showed varying patterns of expression with little consensus between them. This could be due to regulation of the synergism of chitinases as suggested by Gutiérrez-Román et al. (2014); Malinich et al. (2019).

Secondary metabolism related genes continue the trend of increasing expression up to 24 hpi and starkly decreasing at 36 hpi. The fungus may be producing a larger number of secondary metabolites during the early phases of colonization in order to defend against potential competitors or produce signaling molecules required for the establishment of symbiotic interactions. Also, the medium in which the fungus and plants were cultivated favors fungal growth, so the ideal growing conditions may have contributed to production of additional secondary metabolites that were not necessary once internal colonization of the plant had occurred. In addition to the dehydrogenases and cytochrome P450 genes, among the upregulated genes, there were also multicopper oxidases and an amine oxidase that may have contributed to nutrient scavenging (Levasseur et al., 2010).

Defense mechanisms appeared to be largely unchanged throughout the time course, with three identified upregulated DEGs. One of the DEGs was a putative myrosinase precursor. Myrosinases are involved in plant defense against pathogens and herbivores (Szucs et al., 2018). The other two defense-related genes upregulated by the fungus were von Willebrand factor domain proteins. Interestingly, proteins in this family are involved in maintaining the health of insect cuticles (Han et al., 2017). Han et al. claim that these proteins are often targets of entomopathogenic fungi, so perhaps *T. virens* utilizes these proteins to recruit potential entomopathogenic fungi as a source of nutrition.

The lipid transport and metabolism category was examined to potentially find genes associated with plant oxylipin biosynthesis pathways associated with defense and ISR, which was recently shown to pay a major role in *T. virens* interaction with maize (Wang K. et al., 2020). Of potential relevance to the induction of the plant oxylipin synthesis is increase expression of the predictively secreted lysophospholipase and acyl-CoA synthase. However, most of the identified genes appeared to be involved in standard membrane maintenance mechanisms, including a cytochrome P450 gene likely to be involved in ergosterol synthesis (Yoshida, 1988).

A notable gene in the energy production and conversion category was a protein containing a FAD binding domain that was highly upregulated at the very early timepoints (6 and 12 hpi) and then expressed similarly to the wild-type strain afterwards. This gene was likely involved in the metabolism of a simple sugar such as glucose or sucrose, which were both present in the growth medium (Yoshida et al., 2015). A second FAD binding domain gene showed a completely different pattern of expression: downregulation at every time point with a spike at 24 hpi. These two FAD binding domain genes are likely binding different substrates. It is possible that the second protein is binding a compound only found within the maize apoplast.

There are several genes with annotations in the general function and unknown function categories. One of particular interest is a defense-related protein containing SCP domain. Proteins with this domain are involved in human and plant defense against fungi (Darwiche et al., 2017). Specifically in plants, the major defense related gene PR1 is an SCP domain containing protein (Darwiche et al., 2017).

Overall, these data suggest that 24 hpi is a key timepoint in the colonization process and seems to be the point at which symbiosis between the plant and fungus begins and internal expansion by the fungus ceases. Multiple key categories show increasing numbers of upregulated genes up to 24 hpi, after which many genes are downregulated. Heatmap analysis supported this hypothesis, as many of the identified genes showed spikes in either direction of expression at 24 hpi. The next step would be to examine if these same gene patterns exhibited in our hydroponic system are found at any time point in soil systems.

A gene co-expression network was generated from the TMM normalized read counts using Netminer software that had been modified to accept input from HiSAT2. Many coexpression networks are generated using only a single algorithm (Amrine et al., 2015; Sun et al., 2017; Mandal et al., 2020). Netminer is useful as it generates a consensus network from three separate network construction algorithms, thus improving the likelihood of correct correlations.

Principal component analysis (PCA) was utilized to reduce the dimensionality of the dataset from 30 dimensions down to the top three dimensions that account for the largest amount of variance in the dataset. The top three principal components were used to cluster genes into groups that were expressed similarly. This clustering step allowed for additional dimensional reduction by limiting the number of genes that were being analyzed at a time. Each gene in each cluster was queried against the generated network to identify its betweenness centrality. Betweenness centrality is the measure of distance between a node (gene) and every other node in the network. Those with the shortest distance values are assigned a higher score. The genes with the highest score in each cluster were selected as putative enriched targets for future study. This analysis operates on the assumption that a node/gene with a higher centrality score will be more important to the network, and its removal will induce large phenotypic changes. Nearly 40% of the enriched targets identified by this analysis would not have been found using the rack-and-stack method previously employed in our laboratory.

One of the most important targets revealed by this analysis pipeline was 111830 or *Sm2*. This gene has been previously demonstrated as having roles in both colonization and induced systemic resistance, and is a paralog of the well-known elicitor *Sm1* (Crutcher et al., 2015; Gaderer et al., 2015). The identification of *Sm2* as a target by this pipeline demonstrates its effectiveness in enriching target gene selections.

Another promising candidate that has not previously been characterized is 50666. This protein was previously found in maize root apoplastic space, in the same region as Sm1 (Nogueira-Lopez et al., 2018). The algorithmic identification of 50666 as an enriched target and its demonstrated presence in plant apoplastic spaces bolsters the efficacy of the presented pipeline.

Additionally, one-third of the enriched targets were small, secreted cysteine-rich proteins. Most effectors and elicitors share this trait, including SM1 and SM2. The likelihood of the involvement of these types of proteins in plant-microbe interactions is high (Lu and Edwards, 2016; Wang D. et al., 2020). Another common trait in proteins involved in plant-microbe interactions is the carbohydrate binding domain. This analysis revealed a gene (214993) whose only annotation was a carbohydrate binding domain. Many elicitors and effectors contain this domain and, in some instances, cannot function properly without it (Djonović et al., 2006a, 2007; Brotman et al., 2008).

Based on the traits discussed here, this pipeline is a very promising tool for the enrichment of targets involved in plant-microbe interactions. We are currently conducting characterization experiments with several of the genes listed in Table 1 for confirmation of their predicted roles in the *T. virens*-maize interaction.

As strains of *T. virens* are noted for their ability to induce host defense responses, we sought to further analyze a transcriptomic study that incorporated a mutant that lacked the ISR elicitor SM1 and a mutant that enhanced ISR (Δ*Sir1*). Interestingly, *Sir1* mutants showed a significantly larger change in expression patterns compared to *Sm1* mutants. This was unexpected as we had hypothesized that *Sm1* had a larger role than *Sir1* in plant-fungal interactions. Based on this analysis, it would appear that *Sir1* actually regulates much of the fungal response to the presence of the plant. Also, it is possible that many of the DEGs in this mutant were due to overexpression of the *Sm1* gene as shown in Wang K. et al. (2020). Many of the DEGs in the Δ*sir1* mutant were involved in signaling, transport, carbohydrate metabolism, and secondary metabolism. Δ*sm1* displayed significant changes in the expression of downregulated genes at 30 hpi, and intriguingly the expression pattern was similar to that of the Δ*sir1* mutant at 6 hpi. SIR1 protein production has been previously demonstrated to be significantly reduced in the presence of maize roots, and plants treated with the deletion mutants exhibited smaller foliar lesions upon pathogen attack (Lamdan et al., 2015). However, another “sir” group protein, 92810, was found in the apoplast of maize roots (Nogueira-Lopez et al., 2018). This protein had a similar reduction in abundance and mutant phenotype as Δ*sir1* (Lamdan et al., 2015). Based on these similarities, SIR1 may also be present in the apoplast or even translocated into maize cells and unable to be liberated by the methods used in Nogueira-Lopez et al. (2018). Alternatively, SIR1 may remain outside of the plant cell and serve as a MAMP protein, where it can induce a response from the plant causing the cascade of gene expression observed in this study.

Another interesting pattern was observed during heatmap analysis where genes that were up or downregulated at one time point would behave oppositely at the other timepoint (Figure 4). This could be due to the length of time between the time points (6 and 30 hpi). The early period time course demonstrated that much of the interaction occurs between 6 and 24 hpi, with significant changes in expression at 36 hpi. The combination of this dataset with the time course dataset generated in this study would substantially increase the predictive power of the gene co-expression network and target enrichment pipeline.

In conclusion, our results here demonstrate that the early phases of colonization are important for the establishment of symbiosis between the plant and fungus. Additionally, further work is planned to characterize several of the target genes generated from our pipeline, starting with the putative bicupin gene, 50666. These mutants will be characterized for their ability to colonize maize roots and induce systemic resistance against a foliar pathogen. Finally, SIR1 appears to be the major regulator of the fungal response to the presence of a potential host. These findings provide the basis for future studies into the response of *T. virens* when establishing a symbiotic relationship with a potential plant host.

## Data Availability Statement

The datasets presented in this study can be found in online repositories. The names of the repository/repositories and accession number(s) can be found at: NCBI GEO, accession no: GSE181269.

## Author Contributions

JT wrote the manuscript, designed experiments, analyzed data, and conducted experiments. K-DW designed and conducted experiments. JT, BH, MK, and CK conceived the study. BH, MK, and CK supervised the study and secured funding required for completion of the study. All authors contributed to the article and approved the submitted version.

## Conflict of Interest

The authors declare that the research was conducted in the absence of any commercial or financial relationships that could be construed as a potential conflict of interest.

## Publisher's Note

All claims expressed in this article are solely those of the authors and do not necessarily represent those of their affiliated organizations, or those of the publisher, the editors and the reviewers. Any product that may be evaluated in this article, or claim that may be made by its manufacturer, is not guaranteed or endorsed by the publisher.

## Supplementary Material

Supplementary Figure 1 Visualization of the *T. virens* gene co-expression network. Each blue rectangle represents a single gene in the *T. virens* genome. The lines connecting genes, called edges, represent a high likelihood of co-expression.

Supplementary Figure 2 A three-dimensional plot of the top three principal components. Different colors represent different clusters as determined by the DBSCAN algorithm. Many clusters are not visible due to the density of the data points.
