# Supplementary figures and images for: Early Transcriptome Response of Trichoderma virens to Colonization of Maize Roots
Source: Front Fungal Biol. 2021 Aug 25;2:718557. doi: 10.3389/ffunb.2021.718557 (PMC10512331; doi:10.3389/ffunb.2021.718557)

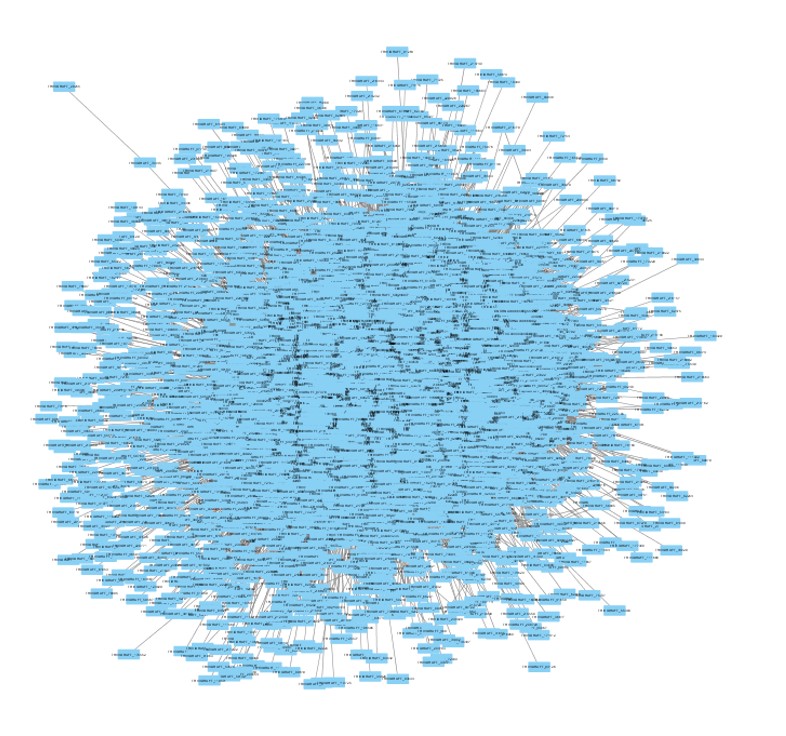

Supplement: Supplementary Figure 1 — Visualization of the T. virens gene co-expression network. Each blue rectangle represents a single gene in the T. virens genome. The lines connecting genes, called edges, represent a high likelihood of co-expression. [file Image_1.JPEG]

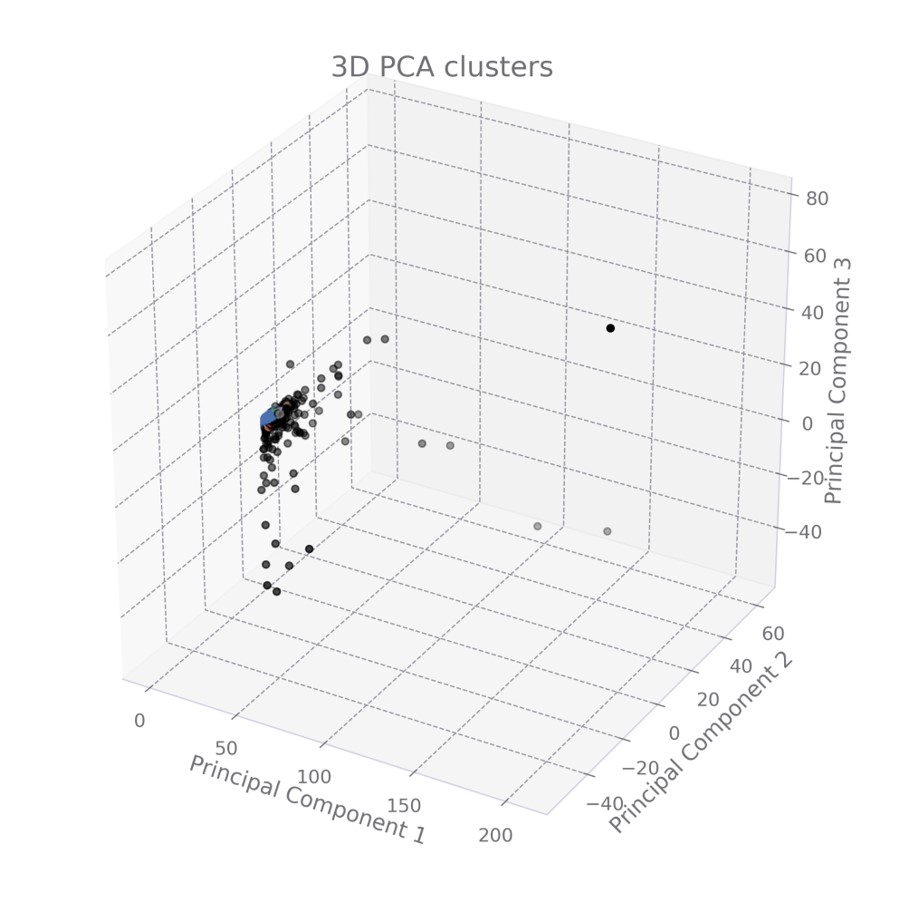

Supplement: Supplementary Figure 2 — A three-dimensional plot of the top three principal components. Different colors represent different clusters as determined by the DBSCAN algorithm. Many clusters are not visible due to the density of the data points. [file Image_2.JPEG]
